# Evolution of intestinal and multivisceral transplantation: A thirty-year United States perspective

**DOI:** 10.1016/j.intf.2024.100022

**Published:** 2024-10-23

**Authors:** Mariya L. Samoylova, Samuel J. Kesseli, Christine Park, John Yerxa, Simon Horslen, Syed-Mohammed Jafri, Alisha Mavis, Thomas Schiano, Bryant Summers, Andrew S. Barbas, Brian I. Shaw, Debra L. Sudan, M. Cristina Segovia

**Affiliations:** a Duke University Hospital, Surgery, United States; b Duke University Hospital, Ped. Gastroenterology, United States; c Seattle Children’s Hospital, Ped. Gastroenterology, United States; d Henry Ford Hospital, Gastroenterology, United States; e Mount Sinai Hospital, Gastroenterology, United States; f Henry Ford Hospital, Transplant Pharmacology, United States; g Duke University Hospital, Gastroenterology, United States

**Keywords:** Intestinal transplantation, Multivisceral transplantation, Outcomes

## Abstract

**Background:**

The field of intestinal transplantation has significantly changed since the report of the first successful transplant in 1988. This report seeks to describe the trends in intestinal transplantation utilization and outcomes over time in the United States of America.

**Methods:**

We use the cohort of intestinal and multivisceral transplants 1990- Feb 2020 in the UNOS STAR dataset. Eras were defined as 1990–1999, 2000–2009, 2010- Feb 2020. Summary statistics were calculated by era. Patient and death-censored graft survival were assessed by era, stratified by pediatric (<18 years at transplant) and adult recipients.

**Results:**

A total of 3035 transplants were performed: 398 in the first era, 1485 in the second, 1235 in the third. The proportion of adult recipients increased over time (35 %, 44 %, 59 % respectively). Fewer livers were included for adults over time (42.8 %, 37.3 %, 36.9 %). One- and five-year patient survival improved over time in children, while adult survival plateaued. A similar trend was observed in death-censored graft survival.

**Conclusions:**

A greater proportion of intestinal transplants are now performed in adults, perhaps as a result of improvements in the intestinal rehabilitation of pediatric patients. Graft and patient survival has improved for pediatric patients but not for adults in the past decade, highlighting the ongoing need for improving long-term outcomes in adult recipients.

## Introduction

Intestinal transplantation (IT) and multi-visceral transplant (MVT) are unique and complex treatments that have evolved considerably in the last 30 years. Since the first reported successful case of isolated intestinal transplantation (IT) in 1988 [1], advances in patient selection, surgical technique, and post-operative management have refined this once experimental operation into a reliable life-sustaining procedure for intestinal failure patients [2], [3], [4], [5]. Despite their life-saving potential, IT and (MVT) remain among the least performed transplants globally, limited in part by the complex logistical coordination required and scarcity of skilled centers.

Currently, IT and MVT are indicated for patients with irreversible causes of intestinal failure or short-gut syndrome and concurrent complications of total parenteral nutrition (TPN) therapy (e.g. line infections, central venous thrombosis, frequent dehydration episodes, or parenteral nutrition associated liver failure) as well as in patients with or without intestinal failure with extensive splanchnic thrombosis or with desmoid tumors suffering from life-threatening complications. While advances in liver-protective TPN formulas and intestinal failure rehabilitation have reduced the incidence of these complications, a subset of patients eventually require transplant referral [6], [7]. Furthemore, although several investigations have demonstrated that IT and (MVT) are cost effective [8], [9] and improve patient quality of life [9], [10], the morbidity of intestinal transplantation should not be understated. The operation is technically complex, often performed in a hostile field with reported mortality due to technical error approaching 6 % [11]; though certain high-volume centers have reported better technical outcomes in selected recipients [12]. Finally, intestinal grafts necessitate high levels of immunosuppression relative to other organ types, making these patients uniquely susceptible to high rates of infection, malignancy, and graft failure. While improving over time, these post-operative issues remain significant barriers in intestinal and multivisceral transplantation. A national examination into the evolution of IT and MVT that led us to this point is currently lacking in the literature.

While others have sought to characterize the single-center experience of IT and MVT recipients [13], [14], [15], these studies highlight variability in experience with IT, especially with respect to inclusion of different parts of the graft and the optimal management of waitlist patients as they focus on patients who underwent transplant. This study sought to use the UNOS STAR file to analyze national trends in the practice and outcomes of IT and MVT over the last three decades in the United States, across the care continuum from listing to post-transplant management.

## Materials and methods

### IRB approval

IRB exemption was obtained and justified by the retrospective nature of the study and anonymization of data, aligning with ethical guidelines for minimal risk studies.

### Data collection and characterization

This study used data from the United Network for Organ Sharing (UNOS) Standard Transplant And Analysis files. We used the cohort of IT and MVT 1990–2020 performed in the United States. Eras were defined as 1990–1999, 2000–2009, 2010- February of 2020. Transplants performed March 1, 2020 and later were excluded to avoid capturing the effect of the COVID-19 pandemic. Free-text diagnosis entries were searched for keywords when the primary diagnosis code was missing or inappropriate (e.g. gastroschisis in an adult). Free-text diagnoses entries for cause of death were searched for keywords when the primary diagnosis code was listed as “missing” or “other.” Lymphocyte-depleting induction” was used to describe induction with anti-thymocyte globulin or alemtuzumab. IL2-R antagonist induction rates were not consistently available.

### Statistical analysis

Summary statistics were calculated by era and comparisons made using appropriate parametric or non-parametric statistical tests. Patient and death-censored graft survival were assessed by either era or inclusion of a liver allograft at prespecified time-points and using Kaplan-Meier (KM) curves. These were stratified by pediatric (<18 years at transplant) and adult recipients. A logrank test was used where appropriate to statistically compare survival curves. Missingness of data is noted in all relevant tables.

## Results

### Overall patient demographics

A total of 5146 patients were registered on the intestinal transplant waiting list during the study period: 787 in the first era, 2359 in the second, and 2000 in the third. Cumulative incidence of transplants by year on waitlist is reported in Table 1; both adult and pediatric transplant rates decreased in the recent era. Waitlist mortality, however, decreased substantially from 2000–2009 to 2010–2020: 17.5 % to 10.4 % for adults, and 24.0 % to 5.8 % for children in the first year since listing (Table 2). The proportion of inactivations for “too sick” decreased in the most recent era, while the proportion of inactivations due to candidate choice increased (Table 3). We also examined the time patients spent on the waitlist prior to transplant or de-listing due to being “too sick”. While median time to transplant among pediatric patients was 116 days (IQR 41–267) and 61 days (IQR 20–155) for adults, median time to inactivation due to sickness was much longer: 279 days (IQR 97–791) for pediatric patients and 206 days (IQR 69–700) for adults. The median age of listing for children increased over time. (Table 4).

**Table 1:** Cumulative incidence of transplant by year on waitlist.

Adults				
	Year 1	Year 2	Year 3	Year 4
1990 −1999	61.1 %	70.8 %	72.1 %	74.0 %
2000 −2009	76.0 %	82.9 %	83.8 %	84.3 %
2010 −2020	69.3 %	78.3 %	82.6 %	83.6 %
**Children**				
	**Year 1**	**Year 2**	**Year 3**	**Year 4**
1990 −1999	46.0 %	59.2 %	64.7 %	68.9 %
2000 −2009	53.8 %	64.6 %	68.3 %	71.9 %
2010 −2020	50.2 %	59.6 %	64.1 %	65.6 %

**Table 2:** Cumulative incidence of death by year on waitlist.

Adults				
	Year 1	Year 2	Year 3	Year 4
1990 −1999	5.0 %	7.7 %	19.5 %	25.1 %
2000 −2009	17.5 %	33.8 %	37.2 %	39.4 %
2010 −2020	10.4 %	18.4 %	22.1 %	32.9 %
**Children**				
	**Year 1**	**Year 2**	**Year 3**	**Year 4**
1990 −1999	3.9 %	6.7 %	8.4 %	10.7 %
2000 −2009	24.0 %	29.5 %	32.2 %	33.2 %
2010 −2020	5.8 %	7.2 %	9.2 %	9.9 %

**Table 3:** Reason for waitlist inactivation.

Adults		
	2000-2009 (n = 345)	2010-2020 (n = 850)
Candidate choice	17 (4.93 %)	130 (15.3 %)
Insurance issues	51 (14.8 %)	91 (10.7 %)
Medical non-compliance	3 (0.9 %)	18 (2.1 %)
Too sick	189 (54.8 %)	399 (46.9 %)
Too well	35 (10.1 %)	51 (6.0 %)
**Children**		
	**2000 −2009** (n = 668)	**2010 −2020** (n = 668)
Candidate choice	70 (10.5 %)	89 (13.3 %)
Insurance issues	23 (3.4 %)	58 (8.7 %)
Medical non-compliance	18 (2.7 %)	28 (4.2 %)
Too sick	232 (34.7 %)	203 (30.4 %)
Too well	293 (43.9 %)	212 (31.7 %)

*Era 1 was not included in this anlaysis as there was no data on reasons for inactivations prior to 2000.

**Table 4:** Patient and transplant characteristics for pediatric intestine and multivisceral transplant recipients.

	1990-1999	2000-2009	2010-2020	p-value
	N = 260	N = 836	N = 498	
**Gender(M)-n(%)**	144 (55.4 %)	453 (54.2 %)	299 (60.0 %)	0.11
**Recipient Age-Med(IQR)**	2.0 (1.0 −6.0)	1.0 (0.0 −4.0)	3.0 (1.0 −7.0)	< 0.001
**Age less than 5?-n(%)**	178 (68.5 %)	647 (77.4 %)	314 (63.1 %)	< 0.001
**Age at Listing-Med(IQR)**^**^**^	1.0 (0.0 −6.0)	1.0 (0.0 −3.0)	2.0 (1.0 −6.0)	< 0.001
**Race-n(%)**				0.001
White	182 (70.0 %)	462 (55.3 %)	265 (53.2 %)	
Black	38 (14.6 %)	167 (20.0 %)	117 (23.5 %)	
Asian	34 (13.1 %)	162 (19.4 %)	92 (18.5 %)	
Native American	0 ( 0.0 %)	20 ( 2.4 %)	14 ( 2.8 %)	
Native Hawaiian/PI	2 ( 0.8 %)	8 ( 1.0 %)	5 ( 1.0 %)	
Multiracial	1 ( 0.4 %)	7 ( 0.8 %)	0 ( 0.0 %)	
Unknown	3 ( 1.2 %)	10 ( 1.2 %)	5 ( 1.0 %)	
**Hispanic?-n(%)**	37 (14.2 %)	166 (19.9 %)	94 (18.9 %)	0.12
Previous Transplant?-n(%)	22 ( 8.5 %)	75 ( 9.0 %)	71 (14.3 %)	0.005
**Organs Included?**				
Duodenum	22 ( 8.5 %)	248 (29.7 %)	171 (34.3 %)	< 0.001
Large Intestine	28 (10.8 %)	98 (11.7 %)	153 (30.7 %)	< 0.001
Small Intestine	236 (90.8 %)	823 (98.4 %)	497 (99.8 %)	< 0.001
Stomach	20 ( 7.7 %)	160 (19.1 %)	127 (25.5 %)	< 0.001
Kidney	11 ( 4.2 %)	25 ( 3.0 %)	19 ( 3.8 %)	0.50
Liver	181 (69.6 %)	593 (70.9 %)	319 (64.1 %)	0.031
Pancreas	42 (16.2 %)	364 (43.5 %)	311 (62.4 %)	< 0.001
**Cause of Death**				0.010
Graft Failure	15 (10.8 %)	20 ( 5.8 %)	5 ( 4.6 %)	
CV Failure	11 ( 7.9 %)	25 ( 7.2 %)	15 (13.8 %)	
Hemorrhage	7 ( 5.0 %)	19 ( 5.5 %)	7 ( 6.4 %)	
Infection	54 (38.8 %)	128 (37.0 %)	28 (25.7 %)	
Multi-Organ Failure	17 (12.2 %)	57 (16.5 %)	17 (15.6 %)	
Malignancy	7 ( 5.0 %)	17 ( 4.9 %)	6 ( 5.5 %)	
CVA	2 ( 1.4 %)	8 ( 2.3 %)	2 ( 1.8 %)	
Respiratory Failure	6 ( 4.3 %)	33 ( 9.5 %)	23 (21.1 %)	
Renal Failure	5 ( 3.6 %)	5 ( 1.4 %)	2 ( 1.8 %)	
GVHD	0 ( 0.0 %)	3 ( 0.9 %)	0 ( 0.0 %)	
Liver Failure	3 ( 2.2 %)	4 ( 1.2 %)	1 ( 0.9 %)	
Unknown	12 ( 8.6 %)	27 ( 7.8 %)	3 ( 2.8 %)	
**Diagnosis at Transplant**				< 0.001
Intestinal Atresia	23 ( 8.8 %)	91 (10.9 %)	44 ( 8.8 %)	
NEC	33 (12.7 %)	135 (16.1 %)	75 (15.1 %)	
Volvulus	54 (20.8 %)	108 (12.9 %)	48 ( 9.6 %)	
Gastroschisis	50 (19.2 %)	209 (25.0 %)	105 (21.1 %)	
Massive Resection	4 ( 1.5 %)	14 ( 1.7 %)	16 ( 3.2 %)	
Other SGS	10 ( 3.8 %)	52 ( 6.2 %)	34 ( 6.8 %)	
Functional Bowel Problems	61 (23.5 %)	165 (19.7 %)	103 (20.7 %)	
Retransplant	12 ( 4.6 %)	42 ( 5.0 %)	52 (10.4 %)	
Other/Unknown	13 ( 5.0 %)	20 ( 2.4 %)	21 ( 4.2 %)	
**Diagnosis of IBD-n(%)**	0	0	0	NA
**Diagnosis of Truama-n(%)**	3(1.2 %)	10(1.2 %)	2(0.40 %)	0.326
**Diagnosis of Thrombosis-n(%)**	2(0.8 %)	8(1.0 %)	18(3.6 %)	0.001
**Depletion Induction Used?-n(%)**^**^**^	3 ( 1.3 %)	217 (26.5 %)	226 (46.4 %)	< 0.001

^< 5 % Missing

### Trends in recipient demographics and immunosuppression

A total of 3118 transplants were performed in the United States during the study period, 398 in the first era, 1485 in the second, 1235 in the third. The proportion of adult recipients increased over time (34.7 %, 43.7 %, 59.7 % respectively). The number of very young child (<5 years) recipients decreased in the last decade (45 %, 44 %, 26 % of all transplants, adult and pediatric). The proportion of transplants performed in non-white recipients increased in both pediatric (30.0 %, 44.7 %, 46.8 %) and adult populations (20.3 %, 15.1 %, 24.4 %), and more transplants were performed in recipients with a previous intestinal transplant over time, particularly among pediatric recipients (9 %, 9 %, 14 %). The proportion of patients receiving T-cell depleting induction immunosuppression regimens has increased over time (Table 4, Table 5).

**Table 5:** Patient and transplant characteristics for adult intestine and multivisceral transplant recipients.

	1990-1999	2000-2009	2010-2020	p-value
	N = 138	N = 649	N = 737	
**Gender(M)-n(%)**	69 (50.0 %)	297 (45.8 %)	347 (47.1 %)	0.65
**Recipient Age-Med(IQR)**	34.0 (28.0 −44.0)	41.0 (30.0 −52.0)	43.0 (31.0 −54.0)	< 0.001
**Age at Listing-Med(IQR)**^	34.0 (28.0 −43.0)	41.0 (30.0 −51.0)	43.0 (31.0 −53.0)	< 0.001
**Race-n(%)**				0.022
White	110 (79.7 %)	551 (84.9 %)	557 (75.6 %)	
Black	13 ( 9.4 %)	53 ( 8.2 %)	91 (12.3 %)	
Asian	10 ( 7.2 %)	36 ( 5.5 %)	71 ( 9.6 %)	
Native American	4 ( 2.9 %)	7 ( 1.1 %)	13 ( 1.8 %)	
Native Hawaiian/PI	1 ( 0.7 %)	1 ( 0.2 %)	2 ( 0.3 %)	
Multiracial	0 ( 0.0 %)	1 ( 0.2 %)	1 ( 0.1 %)	
Unknown	0 ( 0.0 %)	0 ( 0.0 %)	2 ( 0.3 %)	
**Hispanic?-n(%)**	10 ( 7.2 %)	36 ( 5.5 %)	72 ( 9.8 %)	0.012
**Previous Intestinal Transplant-n(%)**	10 ( 7.2 %)	68 (10.5 %)	82 (11.1 %)	0.39
**Organs Included**				
Duodenum	20 (14.5 %)	226 (34.8 %)	298 (40.4 %)	< 0.001
Large Intestine	22 (15.9 %)	57 ( 8.8 %)	226 (30.7 %)	< 0.001
Small Intestine	124 (89.9 %)	642 (98.9 %)	732 (99.3 %)	< 0.001
Stomach	26 (18.8 %)	195 (30.0 %)	275 (37.3 %)	< 0.001
Kidney	7 ( 5.1 %)	46 ( 7.1 %)	55 ( 7.5 %)	0.64
Liver	59 (42.8 %)	242 (37.3 %)	272 (36.9 %)	0.42
Pancreas	33 (23.9 %)	282 (43.5 %)	344 (46.7 %)	< 0.001
**Cause of Death**				0.001
Graft Failure	3 ( 3.6 %)	18 ( 5.2 %)	10 ( 3.6 %)	
CV Failure	9 (10.7 %)	24 ( 6.9 %)	35 (12.5 %)	
Hemorrhage	4 ( 4.8 %)	20 ( 5.8 %)	33 (11.8 %)	
Infection	37 (44.0 %)	120 (34.6 %)	71 (25.4 %)	
Multi-Organ Failure	10 (11.9 %)	36 (10.4 %)	40 (14.3 %)	
Malignancy	6 ( 7.1 %)	27 ( 7.8 %)	14 ( 5.0 %)	
CVA	3 ( 3.6 %)	11 ( 3.2 %)	10 ( 3.6 %)	
Respiratory Failure	2 ( 2.4 %)	28 ( 8.1 %)	28 (10.0 %)	
Renal Failure	0 ( 0.0 %)	5 ( 1.4 %)	1 ( 0.4 %)	
GVHD	0 ( 0.0 %)	1 ( 0.3 %)	5 ( 1.8 %)	
Liver Failure	3 ( 3.6 %)	12 ( 3.5 %)	1 ( 0.4 %)	
Unknown	7 ( 8.3 %)	45 (13.0 %)	31 (11.1 %)	
**Diagnosis at Transplant**				< 0.001
Massive Resection	75 (54.3 %)	264 (40.7 %)	265 (36.0 %)	
Other SGS	14 (10.1 %)	156 (24.0 %)	214 (29.0 %)	
Functional Bowel Problems*	17 (12.3 %)	70 (10.8 %)	80 (10.9 %)	
Retransplant	4 ( 2.9 %)	52 ( 8.0 %)	63 ( 8.5 %)	
Other/Unknown	28 (20.3 %)	107 (16.5 %)	115 (15.6 %)	
**Diagnosis of IBD-n(%)**	4(2.9 %)	25(3.9 %)	25(3.4 %)	0.875
**Diagnosis of Trauma-n(%)**	14(10.1 %)	43(6.6 %)	28(3.8 %)	0.004
**Diagnosis of Thrombosis-n(%)**	30(21.7 %)	182(28.0 %)	177(24 %)	0.13
**Depletion Induction Used?-n(%)**^	0 ( 0.0 %)	340 (53.9 %)	447 (63.2 %)	< 0.001

^< 5 % Missing

* including motility and mucosal disorders

### Trends in transplant indications

The most common indication for intestine-only and multivisceral transplants in adults remained short gut syndrome (65 % in the third era). In children, intestine-only grafts were most commonly used for functional bowel problems (22 % in third era), and multivisceral grafts for gastroschisis (23 % in third era). The relative frequency of necrotizing enterocolitis as the primary diagnosis for transplant in children < 5 years old decreased over time (16.3 %, 11.4 %, 10.0 % for first, second, and third era respectively). Transplants for mesenteric thrombosis and irritable bowel disease (IBD) were stable in adults, while indicatiosn for trauma decreased. There was a slight increase in the rate of thrombosis as an indication for transplant among pediatric patients (Table 4, Table 5).

### Trends in survival and cause of death

**Fig. 1:**
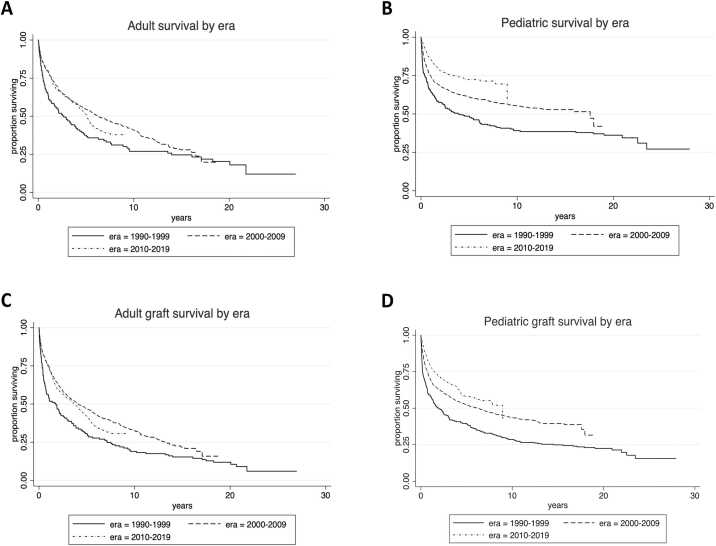
Patient survival after intestinal and multivisceral transplant by era in (A) adults and (B) children. Death-censored graft survival in (C) adults and (D) children.

One- and five-year pediatric survival improved (first era: 64.7 %, 46.0 %; second era 74.35 %, 60.5 %; third era 84.5 %, 72.3 %, respectively; KM log rank era 2 vs. 3 p < 0.0005). One- and five-year adult survival plateaued (first era: 64.7 %, 37.4 %; second era 79.3 %, 53.9 %; third era 77.2 %, 52.2 %, respectively. KM log rank era 2 vs. 3 p = 0.20). A similar trend was observed in graft survival **(**Fig. 1**).** Infection remains the most common cause of death, but is decreasing in relative frequency. Cardiovascular events and respiratory failure as a cause of death have increased in the recent era.

### Trends in liver-inclusive ITs and MVTs

The use of livers in grafts is of special significance. In our national analysis, we found that fewer livers were included in both children and adults but the number of grafts including the duodenum, large intestine, or stomach increased over time. Adult patient survival was worse by point estimate with the inclusion of liver grafts, though this effect was most pronounced and statistically significant only in Era 3. **(**Supplemental Figure 1). Among pediatric patients, a similar trend was seen but was with decreasing effect with era (Supplemental Figure 2). There was no significant effect of liver graft inclusion on adult graft survival in the first two.

eras, though there appeared to be more graft loss with the inclusion of livers in era 3 (Supplemental Figure 3). Among pediatric patients, inclusion of a liver graft appears to have a deleterious effect initially with a mediation of that later in the course, though statistical comparisons using Logrank test cannot be made. **(**Supplemental Figure 4).

## Discussion

Overall, the causes of intestinal failure necesitating IT or MVT have not changed dramatically over the last 30 years, despite introduction of additional (albeit rare) indications [16], [17] for the procedure, but there are several other important findings to highlight. In the last decade, we demonstrate an increase in the proportion of non-White individuals receiving transplants, notable given that non-White race has been associated with increased adjusted mortality and decreased probability of transplant for children < 5years old with intestinal failure [18]. Furthermore, clear improvement has been made in pediatric recipient survival, which now achieves 85 % and 72 % at one- and five-years, respectively, in the most recent era. This has occurred in spite of the fact that the rate of pediatric transplant has decreased overall and pediatric re-transplant rates have increased. In contrast to pediatric patients, graft and patient survival among adults have remained stagnant between the second and third eras with infection remaining the most common cause of death, albeit decreasing relative to other causes. Finally, while waitlist deaths for adults have decreased over consecutive years in the third era compared to the second, the cumulative incidence of death remains unfortunately high at nearly one third of the waitlist dead at four years. The rate of inactivations for candidates being “too sick” for transplant has also remained high. Strategies to encourage timely referral to intestinal rehabilitation and transplantation centers [16], increase the intestinal transplant donor pool [19], and improve upon allocation policy are needed to improve waitlist outcomes for this complex patient population.

There are several potential explanations for our findings. The improved pediatric outcomes are potentially related to advances in multidisciplinary intestinal failure management and patient selection for transplantation [20], [21], [22] but change in surgical technique might also play a role. We noted a greater proportion of pediatric MVT recipients included liver, which has been associated with improved survival in some single center series [23] and is hypothesized to be protective against de novo Donor Specific Antibody (DSA) formation [24], [25], potentially decreasing immunosuppression requirements [26], [27], [28]. There has also been a marked improvement in pediatric waitlist survival, likely due to the development of effective protocols for medically managing intestinal failure [29], [30], [31]. Most of the focus of these protocols has been on achieving adequate infection control, maintaining proper nutritional status, ensuring mobilization, and delivering appropriate wound care [30] and such efforts could have contributed to the improved pediatric waitlist death despite worsening health of the patient population. With regards to the rising transplant rate of non-Whites, while we are encouraged by the point estimates, we must be wary of noting a decrease in disparity without specific information on the denominator of patients eligiable for intestinal transpalnt [32].

We also demonstrate a change in the proportion of colon- and stomach-containing grafts, both of which have increased for both pediatric and adult MVT recipients. Colon and ileocecal valve inclusion have been shown to improve stool formation, continence, quality of life, and rates of stoma closure after transplant with no additional morbidity [33], [34]. A gastric conduit is often included due to institutional preference and in patients with pre-existing gastric or duodenal dilatation secondary to chronic intestinal pseudoobstruction or those with prior gastric resections [35]. Despite this, gastric allograft dysmotility resulting in poor gastric emptying continues to be a nearly universal phenomenon due to graft denervation, but varies in severity after transplant and can be treated with promotility agents [36], [37].

High waiting time continues to be a problem for intestinal transplant patients, with the most recent national report noting that a large plurity of adults patients are on waiting lists for more than 2 years. This squares with our analysis that shows that 1/3 of patients on the waiting list at four years. Though difficult to parse based on imperfect registry data, this could be because patients are slightly sicker at the time of presentation. Age has certainly increased since in the last 20 years and more patients are presenting with previous transplants. Focussing on availability of donors, it shoud be noted that intestines are not recoverable from donation after cardiac death donors due to warm ischemia. In addition, there are technical challenges of vessel length when procuring isolated intestines and liver for two different recipients. Finally, allocation policies which decrease local prioritization of intestine grafts has made it harder to find suitable grafts in some cases, as, again, intestine containing grafts are exceedingly sensitive to ischemia [38].

Relative to other countries, intestinal transplant in the US remains relatively prevalent. Interestingly, small changes in allocation or the culture around the use of TPN appear to have large effects on the use of ITx. In a recent study of the European experience, France has nearly stopped performing intestinal transplant due to an increased reliance on TPN and the UK increased their ITx experience greatly due to a change in allocation. [39] Addtionally, the worldwide data mirror our own in terms of decreased waitlist mortality among pediatric patients especially [16]. As noted above, the improvement in long term management of TPN has likely contributed to both decreased waitlist death as well as a decrease in referral to transplant that is true both in the US and around the world.

In this study, we hoped to summarize the trends in the United States in IT and MVT over the last three decades, highlighting both advancements and persistent challenges in treating this complex patient population, but acknowledge several limitations to this analysis stemming from the use of registry data. In general, our methods are subject to the problems of incomplete or inaccurate registry entry and limitations in analysis due to non-random missingess, (though we favor this to be less likely given our relatively high n in each group). There are specific limitations worthy of consideration. For example, our discussion of pre-2000 transplants is limited by the relatively lower quality of collected data in that time, which does not include the waiting list and may be limited in accuracy. Additionally, our assessment of cause of death in the recent era may be biased towards earlier events due to the available duration of follow-up. We can also not compare the illness or medical acuity of IT or MVT recipients between eras, an important consideration given the improvements in intestinal rehabilitation as mentioned above.

## Conclusion

In conclusion, IT and MVT are life saving surgical options for patients with complications of intestinal failure. Unfortunately, 33 % of adults and 10 % of children on the intestinal transplant waiting list die by four years on the waiting list, and many are inactive due to being too sick for transplant. While pediatric patient and graft survival has improved markedly in the recent decade, adult survival has not. Further work is needed to optimize intestine transplant referral and allocation, and to improve management of immunosuppression to reduce rejection and infection-related mortality.

## Patient’s/guardian’s consent

Not applicable.

## Ethical approval

This study was deemed exempt from approval by the institution’s ethics board as it was performed using de-identified information.

## Funding

The authors have no funding sources to disclose.

## CRediT authorship contribution statement

**Syed-Mohammed Jafri:** Writing – review & editing, Writing – original draft, Supervision, Methodology, Conceptualization. **Alisha Mavis:** Writing – review & editing, Supervision, Methodology. **Thomas Schiano:** Writing – review & editing, Writing – original draft, Methodology, Conceptualization. **Bryant Summers:** Writing – review & editing, Methodology, Conceptualization. **Andrew Barbas:** Writing – review & editing, Writing – original draft, Supervision, Methodology, Conceptualization. **Debra L Sudan:** Writing – review & editing, Methodology. **M. Cristina Segovia:** Writing – review & editing, Writing – original draft, Supervision, Methodology, Conceptualization. **Mariya Samoylova:** Writing – review & editing, Writing – original draft, Supervision, Software, Methodology, Investigation, Formal analysis, Data curation, Conceptualization. **Brian I Shaw:** Writing – review & editing. **Samuel J Kesseli:** Writing – review & editing, Writing – original draft, Methodology, Data curation, Conceptualization. **Christine Park:** Writing – review & editing, Writing – original draft, Formal analysis, Conceptualization. **John Yerxa:** Writing – review & editing, Writing – original draft, Formal analysis, Data curation, Conceptualization. **Simon Horslen:** Writing – review & editing, Supervision, Methodology.

## Declaration of Competing Interest

The authors declare that they have no known competing financial interests or personal relationships that could have appeared to influence the work reported in this paper.

## Footnotes

Appendix A Supplementary data associated with this article can be found in the online version at doi:10.1016/j.intf.2024.100022.
