# Supplementary figures and images for: Evolution of intestinal and multivisceral transplantation: A thirty-year United States perspective
Source: Intest Fail. 2024 Oct 23;2:100022. doi: 10.1016/j.intf.2024.100022 (PMC12851321; doi:10.1016/j.intf.2024.100022)

### Patient Survival (Adult)-Era 1

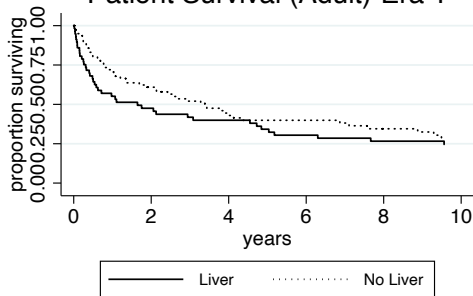

### Patient Survival (Adult)-Era 2

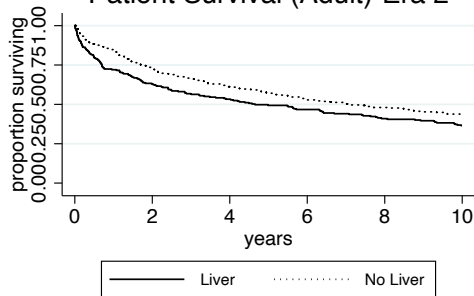

### Patient Survival (Adult)-Era 3

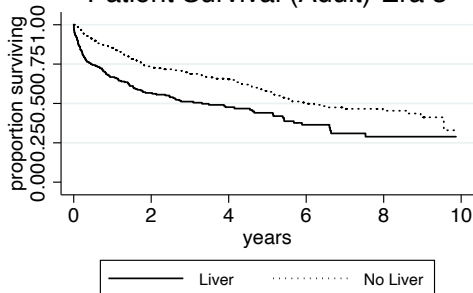

Supplement: Supplementary file 1 — Supplementary material [file mmc1.pdf]

### Patient Survival (Peds)-Era 1

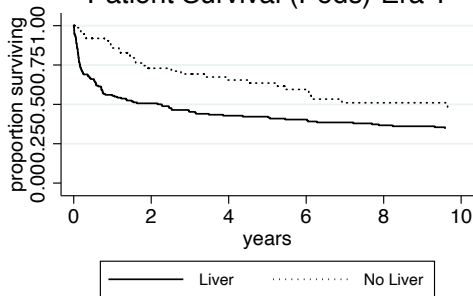

### Patient Survival (Peds)-Era 2

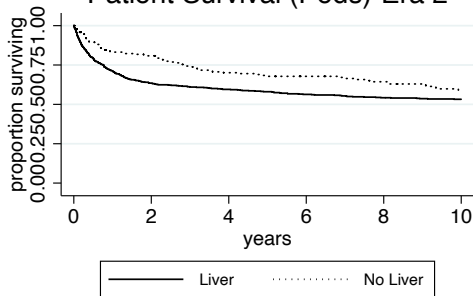

### Patient Survival (Peds)-Era 3

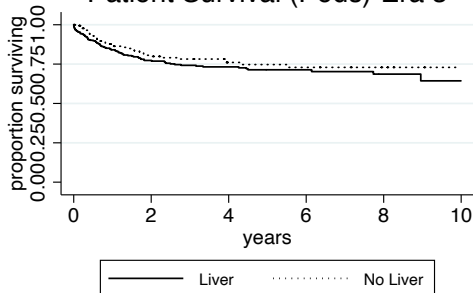

Supplement: Supplementary file 2 — Supplementary material [file mmc2.pdf]

### Graft Survival (Adult)-Era 1

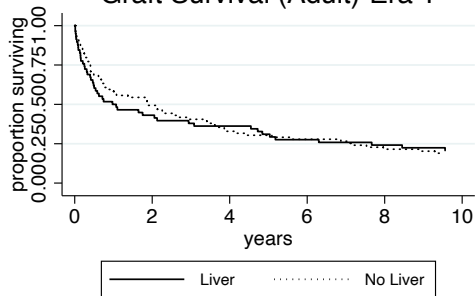

### Graft Survival (Adult)-Era 2

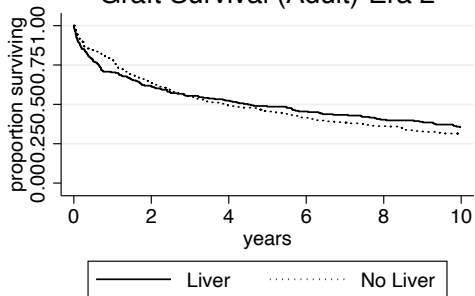

### Graft Survival (Adult)-Era 3

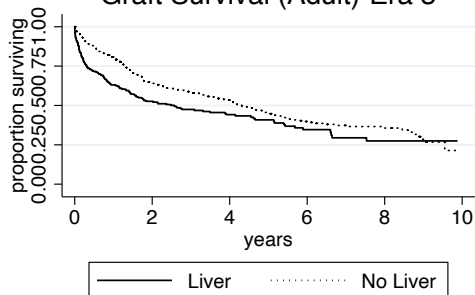

Supplement: Supplementary file 3 — Supplementary material [file mmc3.pdf]

### Graft Survival (Peds)-Era 1

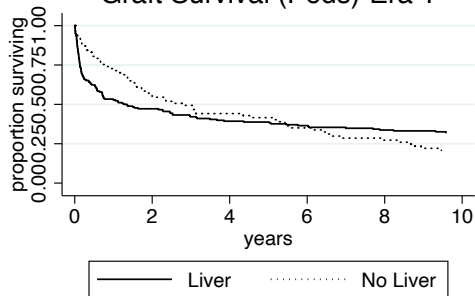

### Graft Survival (Peds)-Era 2

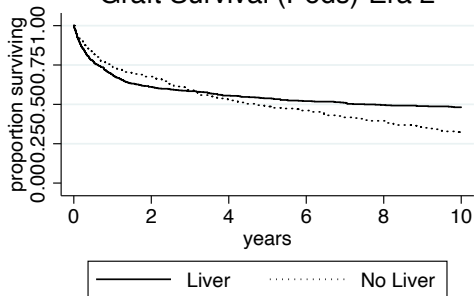

### Graft Survival (Peds)-Era 3

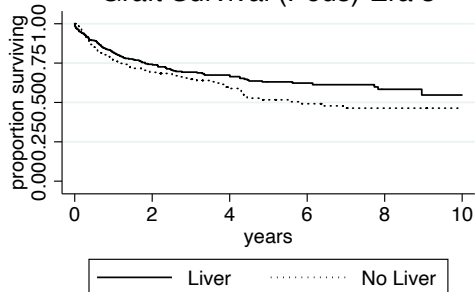

Supplement: Supplementary file 4 — Supplementary material [file mmc4.pdf]
